# Evolution in the surgical management of gastric cancer: is extended lymph node dissection back in vogue in the USA?

**DOI:** 10.1186/s12957-017-1204-6

**Published:** 2017-07-17

**Authors:** Tianxiang Chen, Dongsheng Yan, Zhiqiang Zheng, Jiayi Yang, Xiang Da (Eric) Dong

**Affiliations:** 1 Department of Thoracic Surgery, Shanghai Chest Hospital, Shanghai Jiaotong University, Shanghai, China; 2 School of Ophthalmology and Optometry, Eye Hospital, Wenzhou Medical University, Wenzhou, Zhejiang China; 3 The Second Affiliated Hospital, Wenzhou Medical University, Wenzhou, Zhejiang China; 4 Department of Clinical Medicine, Jiaxing University, Jiaxing, Zhejiang China; 5 Department of Surgery, Westchester Medical Center, 100 Woods Road, Valhalla, NY 10595-1530 USA

**Keywords:** Gastric cancer, D2 lymphadenectomy, Lymph node dissection, Gastrectomy

## Abstract

**Background:**

Gastric cancer remains a formidable treatment challenge. For decades, treatment consisted mostly of surgical intervention for this deadly disease. With improvements in the multi-disciplinary management of solid organ malignancies, the approach to this disease is being stepwise refined.

**Main body:**

One of the prevalent controversies in the surgical management of gastric cancer rests on the need for adequate harvesting of lymph nodes. For decades, lymph node dissection is regarded as a staging technique useful in only upstaging the disease. The adoption of D2 lymphadenectomy has been particularly slow to mature. But with prevailing data from Asia consistently demonstrating a survival benefit from lymphadenectomy, it calls into question the notion of lymphadenectomy as being solely a staging procedure.

**Conclusions:**

As gastric resection techniques are being better defined in western countries and surgical morbidities lowered on its execution, D2 lymphadenectomy is becoming more accepted as the new standard in the management of gastric cancer.

## Background

Gastric cancer is the fifth most common cause of cancer worldwide with an estimated incidence of around 952,000 cases per year [1]. It is the third leading cause of cancer death in both sexes worldwide accounting for 8.8% of the total deaths from all causes of malignancy [1]. There has been a substantial decrease in the incidence of gastric cancer compared to that in 1975 when estimates began being tracked by GLOBOCAN worldwide [1]. With the advent of minimally invasive surgical approaches and targeted chemotherapeutic agents, a shift toward more individualized, stage-dependent treatment of gastric cancer has been advocated, with the intention of achieving better treatment efficacy with less burdensome procedures for patients with either early or advanced diseases. However, current treatment approaches to the management of gastric cancer frequently centered around management of localized diseases, staging, and appropriate adjuvant chemotherapy. General consensus, prevalent in the 1980s and 1990s, about extended lymphadenectomy being a tool for accurate staging of disease without added survival benefit continues to persist. The driving principle that lymph nodes are regarded as indicators rather than governors of disease, as outlined by Dr. Cady, relegated D2 lymphadenectomy in gastric surgery to a staging tool rather than exerting a therapeutic effect [2].

Utilizing extended lymphadenectomy as the prevailing treatment approach, the survival data from Japan and other Asian countries bested the results of surgeons here in the west [3, 4]. Trials in Japan showed consistent overall survival rates in excess of 70% for gastric cancer, a remarkable feat compared to results seen in their western counterparts [5]. Against this backdrop, several trials were undertaken to assess the effectiveness of D2 lymphadenectomy on the western population [6–9]. Both the UK and the Netherlands published results of their much sought-after gastric cancer trials in 1999 [6, 7]. Early results from both trials showed that D1 and D2 lymphadenectomy showed equivalent results for both overall and progression-free survival for patients with resectable gastric cancer [6–9]. The results of these studies seem to support the surgical paradigm that lymphadenectomy is purely a staging rather than a therapeutic tool [10].

Yet over the last two decades, the survival data coming from Asia continues to challenge western practice patterns. The sentiment among the surgical community is that the gastric cancers treated around the world are different entities resulting in the large differences in survival and recurrence patterns [3, 4, 11]. Against this backdrop, several smaller trials continued to chip away at the engrained surgical management plan [12–17]. The Italian trial was undertaken, starting in 1999 and published in 2004, demonstrating that reduced morbidity and mortality in patients undergoing D2 lymphadenectomy is possible in western countries [15]. Later, multiple trials showed that inadequate lymph node harvesting can have deleterious effects on the outcome of the patient [18–23]. Local regional recurrence rates were also alarmingly high in patients following D1 lymphadenectomy with rates as high as 88% following surgery alone [24, 25]. Patients in the Intergroup 0116 trial had a 29% local and 72% regional recurrence rate [26]. For years, the Intergroup 0116 trial supported the use of chemoradiation as adjuvant treatment to improve the survival of patients with gastric cancer [26]. The use of adjuvant radiation hints at the use of radiation to compensate for inadequate local control following less extensive surgical resection [27]. Therefore, current guidelines practiced at major medical centers seem to concur that surgical resection for gastric cancer requires a D2 lymphadenectomy in conjunction with appropriate chemotherapy [28–31].

With rapid changes in management options in the field of gastric cancer, it is interesting to note that we have shifted toward more aggressive local surgical therapy with recommendations for adequate lymph node dissection to mitigate the risks of local recurrence in subset of patients with gastric cancer [28–31]. This review centers on changes in the surgical paradigm for gastric cancer.

### Gastric cancer lymph node stations

**Fig. 1:**
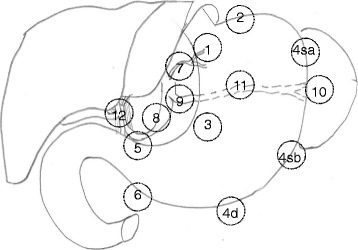
Gastric nodal stations. The stations listed are those relevant for D1 and D2 nodal dissections. *4sa* along the short gastric vessel, *4sb* along the left gastroepiploic vessels, *4d* along the second branch and distal part of the right gastroepiploic artery

Lauren et al. initially described several types of gastric cancer, which included those that spread to involve the entire stomach early on or ones that slowly penetrate the muscular wall of the stomach prior to invasion into adjacent organs [32]. Gastric cancer can spread early to surrounding lymph nodes. The current definition of lymph node stations around the stomach and surgical lymphadenectomy is largely depending on the Japanese literature. The 14th edition of the Japanese Classification of Gastric Cancer categorized a total of 20 nodal stations in the region surrounding the stomach [28, 33]. Although variations exist, only nodal stations 1–16 are frequently discussed in relation to D1, D2, and D3 node dissections. The description of the group 1, group 2, and group 3 nodes with relation to the stomach predated the use of D1, D2, and D3 nodal dissection. The lymph node stations 1–6 were regarded as group 1 nodes (Fig. 1). The second group of lymph nodes centered around the mesenteric vessels, namely, the left gastric, common hepatic, celiac, splenic, and proper hepatic arteries. The third group of nodes are near the major vascular trunks and the retroperitoneum. Because of the need to compare data across the east-west divide, the current AJCC/TNM staging system and the 14th edition of Japanese Classification of Gastric Cancer made strides to categorize and stage patients similarly based on the number of lymph nodes rather than the location of the lymph nodes [30, 33].

Previous editions of the Japanese classification noted the relevance of the location of the nodes in relationship to the primary tumor. Studies have shown that tumors located in particular parts of the stomach rarely metastasized outside their designated drainage pattern. Lymphatic drainage tends to be centripedal toward the celiac trunk. Group 1 lymph node metastasis was considered N1 disease, group 2 lymph node metastasis was considered N2 disease, and group 3 lymph node metastasis was considered N3 disease. Only recently, with intent to merge with conventional American TNM staging, nodal metastases became categorized based on the number of positive lymph nodes. This also conformed to the French and the Italian data suggesting that the number of positive lymph nodes impacted survival [34, 35].

Based on the understanding of lymphatic drainage in gastric cancer, D2 lymphadenectomy was initially introduced to encompass nodes commonly seen in gastric cancer. Early in the 1960s, D2 dissection was introduced and later adopted as standard of care in Japan for management of gastric cancer [5, 13, 36, 37]. Techniques for gastric resection based on location of the tumor would entail specific nodal stations. For the most part, only subtotal and total gastrectomies are routinely performed for cancers of the distal stomach and proximal stomach, respectively. As distal subtotal gastrectomy only entailed part of the stomach, the level 2 lymph nodes are spared during routine distal gastrectomy to avoid devascularizing the proximal stomach. Therefore, a D1 nodal dissection encompassed nodal stations 1, 3, 4, 5, 6, and 7 in distal gastrectomy. For total gastrectomy, nodal stations 1–6 and 7 are resected as part of D1 dissection. For D2 dissections, nodal stations 8 through 12a are commonly removed as well in both distal or total gastrectomy (Table 1).

**Table 1:** Types of lymph node dissections [29, 30] (adapted from Japanese Gastric Cancer Treatment Guidelines)

	Total gastrectomy	Distal/subtotal gastrectomy
D1	1–7	1, 3, 4sb, 4d, 5, 6, 7
D1+	1–7, 8a, 9	1, 3, 4sb, 4d, 5, 6, 7, 8a, 9
D2	1–7, 8a, 9, 11p, 12a	1, 3, 4sb, 4d, 5, 6, 7, 8a, 9, 11p, 12a
D3	1–7, 8a, 9, 11p, 12–14	1, 3, 4sb, 4d, 5, 6, 7, 8a, 9, 11p, 12–14

*4sa* along the short gastric vessel, *4sb* along the left gastroepiploic vessels, *4d* along the second branch and distal part of the right gastroepiploic artery, *8a* anterosuperior group, *8p* posterior group, *11p* along the proximal splenic artery, *11d* along the distal splenic artery

### Differences in the care of gastric cancer

The incidence of gastric cancer is significantly lower in the USA compared with that in the eastern countries. Estimated incidence in the USA totaled 21,000 per year, compared to 82,000 per year in the EU bloc of 28 nations [1, 5, 38]. That is in contrast with 108,000 per year in Japan alone and 460,000 cases per year in China [1, 39, 40]. In the USA, the majority of cases for gastric cancer are treated and operated on at centers that perform fewer than 20 gastrectomies per year. High-volume centers in the USA (>20 cases per year) perform a minority of cases [41, 42]. Between the periods of 1998–2003, analysis of over 50,000 patients with gastric cancer found that surgical mortality for gastric cancer was around 6% nationwide [41]. Majority of patients receive a D1 or less nodal dissection as part of their care. Even over 50% of the patients enrolled in the Intergroup 0116 trial received less than a D1 lymphadenectomy [26]. As a result, local regional recurrence tends to be higher in western patients in comparison to patients in eastern countries. Based on a review of the group of patients treated in 1993 done by the American College of Surgeons, overall survival at 5 years was a paltry 19% with local or regional recurrence at 41% [43]. In the Intergroup study, 29% had local recurrences and 72% had regional recurrences [26].

Overtime, the standard surgical treatment for management of gastric cancer evolved with the available evidence. D2 lymph node dissection was adopted by the Japanese in 1981, followed by the Italian Research Group in 1992 [5, 44–46]. Since then, the majority of European nations have followed suit. The NCCN guidelines in the USA are currently recommending a D1+ or a modified D2 lymph node dissection, with the latter performed by experienced surgeons in high-volume center [47]. Several of the major academic institutions have started recommending D2 lymphadenectomy. With practice pattern changes, major centers increased their proportion of patients to over 80% for those undergoing D2 lymphadenectomy with a median nodal harvesting of over 15 nodes [38].

For years, gastric cancer presented a treatment challenge to the western clinician. Without an adequate screening program similar to the ones adopted in Japan [48], patients typically present with symptoms as the first sign of disease. In Japan, 53% of gastric cancer in Japan is localized at presentation as opposed to only 27% in the USA [3, 4]. In fact, up to 50% of stage IA gastric cancer patients can be treated with endoscopic mucosal resection (EMR) in Japan [49]. Although advocated by some, this procedure currently has limited availability in the USA and other western countries.

Within the USA alone, the incidence of gastric cancer has been declining over the last century. Five year survival rates measured 30.4% based on the latest available SEER database [1]. In fact, typical hospital volumes for treatment of gastric cancer remain below 10 cases per year [41]. Gastric cancer treatment algorithm frequently entails surgical resection followed by chemotherapy and radiation as modeled after the Intergroup 0116 trial study [26]. It remains the mainstay of management approaches practiced by both surgeons and oncologists in community practices.

During the early 2000s, several studies were undertaken to examine this issue of nodal dissection with regard to surgical management of gastric cancer (Table 2). The use of D2 lymph node dissection was firmly established in Asian countries, and studies utilizing anything less than D2 dissection were not well accepted [28, 50, 51]. Nodal dissection was seen more than just a proper staging but actual part of the treatment for gastric cancer to minimize loco-regional recurrence. In some instances, surgeons also undertook D3 nodal dissection in patients with gastric cancer in an effort to achieve negative local disease and to mitigate risks of local regional recurrence [12, 14]. These approaches clearly relied on the notion that certain gastric cancer spread in a Halstedian fashion. It is locally aggressive and spreads through a pre-determined nodal drainage pattern and, eventually, via blood-borne metastases [2]. The role of D2 dissection would have become irrelevant if not for the persistent discrepancy in survival data compared with the Asian literature.

**Table 2:** Randomized controlled trials comparing D1 with D2 nodal dissection [43]

Study	Country	Comparison	Postoperative morbidity	Postoperative mortality	5-year survival
Dent et al. (1982–1985)	South Africa	D1 (*n* = 22)	22%	0%	69%
		D2 (*n* = 21)	43%	0%	67%
Bonenkamp et al. (1989–1993)	Netherlands	D1 (*n* = 380)	25%	4%	45%
		D2 (*n* = 331)	43%	10%	47%
			(*P* < 0.001)	(*P* = 0.004)	HR 1.00 (95%CI, 0.82–1.22)
Cuschieri et al. (1987–1994)	UK	D1 (*n* = 200)	28%	6.5%	35%
		D2 (*n* = 200)	46%	13%	33%
			(*P* < 0.001)	(*P* = 0.04)	HR 1.10 (95%CI, 0.87–1.39)
Degiuli et al. (1999–2002)	Italy	D1 (*n* = 76)	10.5%	1.3%	
		D2 (*n* = 86)	16.3%	0%	
			(*P* < 0.029)	(n.s.)	
Degiuli et al. (1998–2005)	Italy	D1 (*n* = 133)	12%	3.0%	66.5
		D2 (*n* = 134)	17.9%	2.2%	64.2
			(*P* = 0.183)	(*P* = 0.725)	(*P* = 0.695)

### Trials on D2 lymphadenectomy

The first trials performed to establish the efficacy of D2 lymphadenectomy were the Dutch and UK trials. The multi-center randomized controlled trial performed in the Netherlands was one of the often analyzed trials undertaken by western countries on gastric cancer [8, 9]. The study was performed at 80 Dutch hospitals with the surgical treatment being performed by 11 specially trained surgeons [8, 9]. A total of 711 patients with curable gastric cancer underwent surgery [8, 9]. Accrual lasted from 1989 to 1993 [8, 9]. Morbidity for D1 and D2 dissections was 25 and 43%, while mortality for D1 and D2 dissections was 4 and 10%, respectively [8, 9]. Five-year follow-up showed that D1 and D2 dissections led to survival of 45 and 47%, which was statistically non-significant [8, 9]. Recently, in the 15-year follow-up analysis, it was shown that D2 lymphadenectomy was associated with lower loco-regional recurrence and gastric cancer-related death rates compared with D1 dissection [8, 9].

Around the same time as the Dutch trial, the Medical Research Council (MRC) conducted a large randomized controlled trial in the UK examining the same issue [7]. A total of 400 patients were randomized to D1 and D2 dissections by one of 32 participating surgeons [7]. Patients with antral tumors underwent distal gastrectomy, while those with middle or upper third lesions underwent total gastrectomy. D1 dissection was defined as removal of lymph nodes within 3 cm of the tumor along with omentectomy [7]. Patients undergoing D2 dissection had their omental bursa and the hepatoduodenal and retroduodenal lymph nodes resected. For upper lesions, splenic hilar nodes and retropancreatic nodes were removed by distal pancreatectomy with splenectomy. Unfortunately, overall survival at 5 years were not different at 35% for D1 and 33% for D2 dissection. As shown later on, the performance of splenectomy likely suppressed the added benefit of a D2 dissection. The authors concluded that patients who underwent a splenic and pancreas-sparing D2 dissection had a better survival than the corresponding D1 group [7].

When the two trials were examined in detail, criticisms of the trials centered on the high morbidity and mortality are seen with D1 and D2 dissection patients [6–9]. Analysis of the causes for the increased morbidity and mortality in the Dutch trial revealed that distal pancreatectomy and splenectomy had a significant detrimental effect on patients. The increase in morbidity and mortality may have affected the final results of the Dutch study since subgroup analysis excluding patients who underwent a pancreatico-splenectomy showed significant survival advantage for those with a D2 lymph node dissection [52, 53]. Three separate studies had been undertaken to look into the effects of splenectomy for gastric cancer [54–56]. Patients with positive metastases in the splenic hilum, which would mandate splenectomy, had poor overall outcome from the onset. Based on the results of those studies, the increased morbidity suggested by some of the studies indicate that splenectomy cannot be recommended at this time [54–56]. Furthermore, an Italian study was then undertaken under strict surgical guidelines to demonstrate that low morbidity and mortality can be achieved in patients undergoing gastrectomy [15, 52, 53]. Part of the survival improvements appear to be related to the preservation of the pancreatic tail and the spleen. Extensive training of the involved physicians also likely contributed to the low morbidity and mortality seen with the Italian study [52, 53].

### Number of lymph nodes impacting survival

The total number of lymph nodes resected, or the total number of positive lymph nodes, or the total number of positive to negative ratio of lymph nodes (nodal ratio) have all been found to be predictors of gastric cancer survival [21, 22, 45]. For potentially resectable gastric cancer ranging from T1/2N0 to T3N1, a linear trend toward superior survival was found for higher lymph node removal up to 40 lymph nodes, based on analysis of the SEER database from 1973 to 1999 [20]. Schwarz et al. reviewed the SEER database for gastric cancer with advanced but potentially curable disease [57]. Patients with node-positive gastric cancer were analyzed for incidence of recurrent disease after resection [57]. The overall survival is improved for every 10 extra LNs added to the total LN count. Therefore, based on the SEER database, stage-based survival was dependent on the total LN number and the number of negative LNs [57].

A higher total harvested negative nodes or lower ratio of positive nodes, referred to as the Maruyama index, also saw improvements in both survival and progression free survival [5, 19, 58]. The Maruyama index was created to determine unresected disease as the sum of regional nodal disease, with higher value on the index portending poorer outcome [5, 19, 58].

In order to improve the number of lymph nodes harvested during surgical dissection, one of the changes that need to occur is the communication between that of the surgeon and the pathologist. Based on studies of specimen handling, when specimens are properly labeled and processed prior to submission for pathological evaluation, nodal counts increased in this process.

On average, within the Dutch trial, the number of patient who actually underwent a proper D2 dissection was 18.4% [27]. Major noncompliance in D2 lymphadenectomy occurred in 26% of the patients [27]. When D2 compliant patients were analyzed alone and compared with D1 group of patients, survival was statistically significant at 35.7 versus 19.9% (*P* = 0.041) [27]. In addition, those that underwent D1 dissection had a sub-D1 dissection in a majority of times. Subgroup analysis did show that patients with D2 dissection had improved local regional control in the long run [16, 17].

The National Comprehensive Cancer Network (NCCN) updated and released their latest guideline in 2010 making changes in their recommendations in terms of the need for nodal dissection. The current recommendations of harvesting a minimum of 15 lymph nodes would satisfy the staging purposes including the category of N3 disease [40, 59]. However, for practical purposes, a minimum of 25 LNs resected would be better during LN dissection [40, 59].

### Survival differences

One of the leading arguments for the comparatively worse outcomes for patients with gastric cancer in western countries has more to do with their stage at diagnosis, rather than the utility of lymphadenectomy [3, 13, 29, 34, 35]. The stage of patients diagnosed with gastric cancer has always been significantly worse in the USA compared with that in Asia [3, 13, 29, 34, 35]. Estimates place limited local regional disease at 53% in Japan versus 27% in the USA at diagnosis [3, 13, 29, 34, 35].

Evaluation of gastric biopsies by pathologists suggests that Japanese pathologists are more likely to consider cellular and glandular abnormalities as diagnostic of carcinoma while western pathologists require presence of tumor invasion [34, 35]. Timing for diagnosis of gastric cancer is therefore considerably later in the western patients compared to that in their eastern cohort. The age of patients at the time of diagnosis is therefore about 10 years older in comparing the two cohorts [34, 35].

Although stage migration alone cannot alone explain the differences in survival, there is perhaps the biology of the tumor in eastern countries [58]. When D2 dissection is carried out in the USA, comparison of patients to their Japanese and Korean cohorts initially showed similar survival to Japanese patients when controlled for tumor location [3, 59]. However, on closer inspection, there is better disease-specific survival with Korean patient when matched stage for stage. Moreover, patients with earlier stage tumors (T1 and T2) in the gastric body had improved survival in the Japanese cohort. Both of these comparisons suggest a difference in the biology of the tumor in the Asian population.

Despite the differences in the two patient cohorts, lymphadenectomy in gastric cancer potentially leads to improved outcome based on three goals in care. The purpose of lymphadenectomy can improve the staging of the disease, prevent the development of loco-regional recurrence, and potentially result in better long-term outcome for the patient. The data so far have been inadequate to completely address the issue in a satisfactory fashion. Studies designed to analyze the utility of D2 lymphadenectomy were performed prior to the general adaptation of standardized techniques in western countries. The results of D2 lymphadenectomy are so well accepted in Asian countries that comparing D1 and D2 dissections in Asia is thought to be below the standard of care [29, 39]. Therefore, while those countries experience the most disease, there is a lack of interest to compare D1 and D2 dissections in the current setting in Asian countries.

On average, as we move across the Atlantic from the USA, to Europe, and finally to Asian countries, lymphadenectomy becomes more and more extensive. Without proper comparative studies inclusive of standardized staging systems, operative techniques, and acceptable morbidity and mortality rates, it has been difficult to produce definitive evidence of survival advantages using D2 lymphadenectomy. Meta-analyses of the limited number of randomized controlled trials are some of the best evidence to date. Using eight randomized controlled trials, Mocellin et al. compared the types of lymphadenectomies on overall survival and disease-specific survival [60, 61]. Their conclusions support the superiority of D2 versus D1 lymphadenectomy in terms of survival benefit, albeit with a moderate level of evidence and with the advantage mainly limited to disease-specific survival (DSS) [60, 61]. More recently, Randle et al. reviewed the US Gastric Cancer Collaborative database and compared patients receiving either D1 or D2 lymphadenectomy [62]. As the patient population was somewhat heterogeneous, they found that patients actually had lower mortality with D2 lymphadenectomy. There was also improved median OS in stage I and III patients [62]. From the Dutch trial results, subgroup analysis showed that survival for the group of patients undergoing D2 dissection without splenectomy was 47 versus 33% for D1 dissection [9]. One of the conclusions was that safer splenic-preserving D2 dissection, when applied in experienced centers, should be the preferred approach in patients with resectable cancer [60, 61].

## Conclusions

The surgical management of gastric cancer have seen the pendulum swing back and forth between aggressive lymph node dissection versus limited nodal sampling. Traditionally, D2 dissection is generally well accepted in Asian countries, whereas a D1 dissection has been well established in western countries until recently. In spite of the multiple studies performed to evaluate gastric cancer lymph node sampling/dissection, there is now begrudging acceptance among the surgical community that gastric cancer nodal dissection is required for good surgical care of patients with gastric cancer. Unfortunately, earlier studies of gastric cancer management with D2 nodal dissection had high morbidity and mortality of patients. More recent long-term analysis of patients undergoing gastric cancer nodal dissection showed that it contributed between 3 and 6% absolute survival benefit in patients with intermediate-stage gastric cancer [61]. It behooves us to look at our practice pattern and improve our surgical care. In 2010, the NCCN guideline committee members updated the recommendations of the committee and added that removal of the nodes along the named vessels of the celiac axis (D2) be performed for resectable tumors [47]. This represented a major shift in the recommendation of the committee as well as the understanding of gastric cancer. For years, after the early data were published regarding the Dutch trials, nodal dissection was thought to lead to stage migration as well as for proper staging of patients. The idea that nodal dissection contributed to the survival of patients still took time to cement their footing in the community. Currently, a proper nodal dissection in the USA is only performed on approximately 30% of patients [41]. There are persistent discussions centering on nodal dissection as a lymph node sampling procedure despite the data in the literature. With the advent and safety of both laparoscopic and robotic surgeries being established in gastrectomies, it is imperative that we get back to the basics. Adequate nodal dissection to achieve accurate staging and proper treatment of patients with the formidable disease can lead to optimal survival.

## Abbreviations

AJCC: American Joint Committee on Cancer
DSS: Disease-specific survival
EMR: Endoscopic mucosal resection
EU: European Union
LN: Lymph ode
MRC: Medical Research Council
NCCN: National Comprehensive Cancer Network
SEER: Surveillance, Epidemiology, and End Results Program

## References

[1] Siegel R, Naishadham D, Jemal A (2012). Cancer statistics, 2012. CA Cancer J Clin..

[2] Cady B (1984). Lymph node metastases—indicators, but not governors of survival. Arch Surg..

[3] Strong VE, Song KY, Park CH, Jacks LM, Gonen M, Shah M (2010). Comparison of gastric cancer survival following R0 resection in the United States and Korea using an internationally validated nomogram. Ann Surg.

[4] Bickenbach K, Strong VE (2012). Comparisons of gastric cancer treatments: east vs. west. J Gastric Cancer.

[5] Maruyama K, Kaminishi M, Hayashi K, Isobe Y, Honda I, Katai H (2006). Gastric cancer treated in 1991 in Japan: data analysis of nationwide registry. Gastric Cancer.

[6] Bonenkamp JJ, Hermans J, Sasako M, van de Velde CJ, Welvaart K, Songun I (1999). Extended lymph-node dissection for gastric cancer. N Engl J Med.

[7] Cuschieri A, Weeden S, Fielding J, Bancewicz J, Craven J, Joypaul V, et al. Patient survival after D1 and D2 resections for gastric cancer: long-term results of the MRC randomized surgical trial. Surgical Co-operative Group. Br J Cancer 1999;79:1522-1530. 10.1038/sj.bjc.6690243 PMC2362742 10188901

[8] Bonenkamp JJ, Songun I, Hermans J, Sasako M, Welvaart K, Plukker JT (1995). Randomised comparison of morbidity after D1 and D2 dissection for gastric cancer in 996 Dutch patients. Lancet.

[9] Hartgrink HH, van de Velde CJ, Putter H, Bonenkamp JJ, Klein Kranenbarg E, Songun I (2004). Extended lymph node dissection for gastric cancer: who may benefit? Final results of the randomized Dutch gastric cancer group trial. J Clin Oncol.

[10] Petrelli N (2004). The debate is over: it’s time to move on. J Clin Oncol.

[11] Wang J, Dang P, Raut CP, Pandalai PK, Maduekwe UN, Rattner DW (2012). Comparison of a lymph node ratio-based staging system with the 7th AJCC system for gastric cancer: analysis of 18,043 patients from the SEER database. Ann Surg.

[12] Sasako M, Sano T, Yamamoto S, Kurokawa Y, Nashimoto A, Kurita A (2008). D2 lymphadenectomy alone or with para-aortic nodal dissection for gastric cancer. N Engl J Med.

[13] Schmidt B, Yoon SS (2013). D1 versus D2 lymphadenectomy for gastric cancer. J Surg Oncol.

[14] Wu CW, Hsiung CA, Lo SS, Hsieh MC, Chen JH, Li AF (2006). Nodal dissection for patients with gastric cancer: a randomised controlled trial. Lancet Oncol.

[15] Degiuli M, Sasako M, Ponti A (2010). Morbidity and mortality in the Italian Gastric Cancer Study Group randomized clinical trial of D1 versus D2 resection for gastric cancer. Br J Surg.

[16] Songun I, van de Velde CJ (2009). Optimal surgery for advanced gastric cancer. Expert Rev Anticancer Ther.

[17] Songun I, Putter H, Kranenbarg EM, Sasako M, van de Velde CJ (2010). Surgical treatment of gastric cancer: 15-year follow-up results of the randomised nationwide Dutch D1D2 trial. Lancet Oncol.

[18] Bouvier AM, Haas O, Piard F, Roignot P, Bonithon-Kopp C, Faivre J (2002). How many nodes must be examined to accurately stage gastric carcinomas? Results from a population based study. Cancer.

[19] Hundahl SA, Peeters KC, Kranenbarg EK, Hartgrink H, van de Velde CJ (2007). Improved regional control and survival with “low Maruyama Index” surgery in gastric cancer: autopsy findings from the Dutch D1-D2 trial. Gastric Cancer.

[20] Smith DD, Schwarz RR, Schwarz RE (2005). Impact of total lymph node count on staging and survival after gastrectomy for gastric cancer: data from a large US-population database. J Clin Oncol.

[21] Schwarz RE, Smith DD. Clinical impact of lymphadenectomy extent in resectable gastric cancer of advanced stage. Ann Surg Oncol. 2007;14:317–328. 10.1245/s10434-006-9218-2 17094022

[22] Chen S, Zhao BW, Li YF, Feng XY, Sun XW, Li W (2012). The prognostic value of harvested lymph nodes and the metastatic lymph node ratio for gastric cancer patients: results of a study of 1,101 patients. Plos One.

[23] Bunt AM, Hermans J, Smit VT, van de Velde CJ, Fleuren GJ, Bruijn JA (1995). Surgical/pathologic-stage migration confounds comparisons of gastric cancer survival rates between Japan and Western countries. J Clin Oncol.

[24] Shen Z, Ye Y, Xie Q, Liang B, Jiang K, Wang S (2015). Effect of the number of lymph nodes harvested on the long-term survival of gastric cancer patients according to tumor stage and location: a 12-year study of 1,637 cases. Am J Surg.

[25] Gunderson LL, Sosin H (1982). Adenocarcinoma of the stomach: areas of failure in a re-operation series (second or symptomatic look) clinicopathologic correlation and implications for adjuvant therapy. Int J Radiat Oncol Biol Phy.

[26] Macdonald JS, Smalley SR, Benedetti J, Hundahl SA, Estes NC, Stemmermann GN (2001). Chemoradiotherapy after surgery compared with surgery alone for adenocarcinoma of the stomach of gastroesophageal junction. N Engl J Med.

[27] de Steur WO, Hartgrink HH, Dikken JL, Putter H, van de Velder CJ (2015). Quality control of lymph node dissection in the Dutch Gastric Cancer Trial. Br J Surg.

[28] de Steur WO, Dikken JL, Hartgrink HH (2013). Lymph node dissection in resectable advanced gastric cancer. Dig Surg.

[29] Yoon SS, Yang HK (2009). Lymphadenectomy for gastric adenocarcinoma: should west meet east?. Oncologist.

[30] Yarema R, de Manzoni G, Fetsych T, Ohorchak M, Pliatsko M, Bencivenga M (2016). On the road to standardization of D2 lymph node dissection in a European population of patients with gastric cancer. World J Gastrointest Oncol.

[31] Roviello F, Marrelli D, Morgagni P, de Manzoni G, Di Leo A, Vindigni C (2002). Survival benefit of extended D2 lymphadenectomy in gastric cancer with involvement of second level lymph nodes: a longitudinal multicenter study. Ann Surg Oncol.

[32] Lauren P (1965). The two histological main types of gastric carcinoma: diffuse and so-called intestinal-type carcinoma: an attempt at a histo-clinical classification. Acta Pathol Microbiol Scand.

[33] Tamura S, Takeno A, Miki H (2011). Lymph node dissection in curative gastrectomy for advanced gastric cancer. Int J Surg Oncol.

[34] Borie F, Plaisant N, Millat B, Hay JM, Fagniez PL (2004). French Association for Surgical Research. Appropriate gastric resection with lymph node dissection for early gastric cancer. Ann Surg Oncol.

[35] Marchet A, Mocellin S, Ambrosi A, Morgagni P, Garcea D, Marrelli D (2007). The ratio between metastatic and examined lymph nodes (N ratio) is an independent prognostic factor in gastric cancer regardless of the type of lymphadenectomy: results from an Italian multicentric study in 1853 patients. Ann Surg.

[36] Strong VE, Yoon SS (2013). Extended lymphadenectomy in gastric cancer is debatable. World J Surg.

[37] van de Velde CJ, Peeters KC (2003). The gastric cancer treatment controversy. J Clin Oncol.

[38] D'Angelica M, Gonen M, Brennan M, Turnbull AD, Bains M, Karpeh MS (2004). Patterns of initial recurrence in completely resected gastric adenocarcinoma. Ann Surg.

[39] Lin Y, Ueda J, Kikuchi S, Totsuka Y, Wei WQ, Qiao YL (2011). Comparative epidemiology of gastric cancer between Japan and China. World J Gastroenterol.

[40] Verlato G, Giacopuzzi S, Bencivenga M, Morgagni P, De Manzoni G (2014). Problems faced by evidence-based medicine in evaluating lymphadenectomy for gastric cancer. World J Gastroenterol.

[41] Enzinger PC, Benedetti JK, Meyerhardt JA, McCoy S, Hundahl SA, Macdonald JS (2007). Impact of hospital volume on recurrence and survival after surgery for gastric cancer. Ann Surg.

[42] Birkmeyer JD, Siewers AE, Finlayson EV, Stukel TA, Lucas FL, Batista I (2002). Hospital volume and surgical mortality in the United States. N Engl J Med.

[43] Wanebo HJ, Kennedy BJ, Chmiel J, Steele G, Winchester D, Osteen R (1993). Cancer of the stomach. A patient care study by the American College of Surgeons. Ann Surg.

[44] Waddell T, Verheij M, Allum W, Cunningham D, Cervantes A, Arnold D (2013). Gastric cancer: ESMO-ESSO-ESTRO clinical practice guidelines for diagnosis, treatment and follow-up. Ann Oncol.

[45] Song W, Yuan Y, Wang L, He W, Zhang X, Chen C (2014). The prognostic value of lymph node dissection number on survival of patients with lymph node-negative gastric cancer. Gastroenterol Res Pract.

[46] Siewert JR, Bottcher K, Stein HJ, Roder JD (1998). Relevant prognostic factors in gastric cancer: ten-year results of the German Gastric Cancer Study. Ann Surg.

[47] Ajani JA, Bentrem DJ, Besh S, D'Amico TA, Das P, Denlinger C (2013). Gastric cancer, version 2.2013: featured update to the NCCN Guidelines. J Natl Compr Canc Netw.

[48] Hanazaki K, Wakabayashi M, Sodeyama H, Makiuchi A, Igarashi J, Yokoyama S (1997). Surgical outcome in early gastric cancer with lymph node metastasis. Hepatogastroenterology.

[49] Uedo N, Iishi H, Tatsuta M, Ishihara R, Higashino K, Takeuchi Y (2006). Longterm outcomes after endoscopic mucosal resection for early gastric cancer. Gastric Cancer.

[50] Nakajima T, Kinoshita T, Nashimoto A, Sairenji M, Yamaguchi T, Sakamoto J (2007). Randomized controlled trial of adjuvant uracil-tegafur vesus surgery alone for serosa-negative, locally advanced gastric cancer. Br J Surg.

[51] Sasako M, Sakuramoto S, Katai H, Kinoshita T, Furukawa H, Yamaguchi T (2011). Five-year outcomes of a randomized phase III trial comparing adjuvant chemotherapy with S-1 versus surgery alone in stage II or III gastric cancer. J Clin Oncol.

[52] Degiuli M, Sasako M, Ponti A, Soldati T, Danese F, Calvo F (1998). Morbidity and mortality after D2 gastrectomy for gastric cancer: results of the Italian Gastric Cancer Study Group prospective multicenter surgical study. J Clin Oncol.

[53] Degiuli M, Sasako M, Calgaro M, Garino M, Rebecchi F, Mineccia M (2004). Morbidity and mortality after D1 and D2 gastrectomy for cancer: interim analysis of the Italian Gastric Cancer Study Group (IGCSG) randomised surgical trial. Eur J Surg Onc.

[54] Furukawa H, Hiratsuka M, Ishikawa O, Ikeda M, Imamura H, Masutani S (2000). Total gastrectomy with dissection of lymph nodes along the splenic artery: a pancreas-preserving method. Ann Surg Oncol.

[55] Csendes A, Burdiles P, Rojas J, Braghetto I, Diaz JC, Maluenda F (2002). A prospective randomized study comparing D2 total gastrectomy versus D2 total gastrectomy plus splenectomy in 187 patients with gastric carcinoma. Surgery.

[56] Yu W, Choi GS, Chung HY (2006). Randomized clinical trial of splenectomy versus splenic preservation in patients with proximal gastric cancer. Br J Surg.

[57] Schwarz RE, Smith DD (2007). Clinical impact of lymphadenectomy extent in resectable gastric cancer of advanced stage. Ann Surg Oncol.

[58] Lu J, Wang W, Zheng CH, Fang C, Li P, Xie JW (2017). Influence of total lymph node count on staging and survival after gastrectomy for gastric cancer: an analysis from a two-institution database in China. Ann Surg Oncol.

[59] Karpeh MS, Leon L, Klimstra D, Brennan MF (2000). Lymph node staging in gastric cancer: is location more important than number? An analysis of 1,038 patients. Ann Surg.

[60] Mocellin S, McCulloch P, Kazi H, Gama-Rodrigues JJ, Yuan Y, Nitti D (2015). Extent of lymph node dissection for adenocarcinoma of the stomach (review). Cochrane Database Syst Rev.

[61] Mocellin S, Nitti D (2015). Lymphadenectomy extent and survival of patients with gastric carcinoma: a systematic review and meta-analysis of time-to-event data from randomized trials. Cancer Treat Rev.

[62] Randle RW, Swords DS, Levine EA, Fino NF, Squires MH, Poultsides G (2016). Optimal extent of lymphadenectomy for gastric adenocarcinoma: a 7-institution study of the U.S. gastric cancer collaborative. J Surg Oncol.

